# Determining Risk for Depression among Older People Residing in Vietnamese Rural Settings

**DOI:** 10.3390/ijerph16152654

**Published:** 2019-07-25

**Authors:** Huyen Thi Thanh Vu, Valentina Lin, Thang Pham, Tuan Le Pham, Anh Trung Nguyen, Hung Trong Nguyen, Thanh Xuan Nguyen, Tam Ngoc Nguyen, Huong Thu Thi Nguyen, Thu Thi Hoai Nguyen, Long Hoang Nguyen, Quang Nhat Nguyen, Huong Lan Thi Nguyen, Tung Hoang Tran, Bach Xuan Tran, Carl A. Latkin, Cyrus S. H. Ho, Roger C. M. Ho

**Affiliations:** 1 Department of Gerontology and Geriatrics, Hanoi Medical University, Hanoi 100000, Vietnam; 2 Scientific Research Department, National Geriatric Hospital, Hanoi 100000, Vietnam; 3 Department of Health Policy and Management, University of Minnesota School of Public Health, Minneapolis, MN 55455, USA; 4 Department of Family Medicine, Hanoi Medical University, Hanoi 100000, Vietnam; 5 Department of Neurology, Hanoi Medical University, Hanoi 100000, Vietnam; 6 Dinh Tien Hoang Institute of Medicine, Hanoi 100000, Vietnam; 7 Center of Excellence in Behavioral Medicine, Nguyen Tat Thanh University, Ho Chi Minh City 700000, Vietnam; 8 Université Claude Bernard Lyon 1, 69100 Villeurbanne, France; 9 Center of Excellence in Evidence-based Medicine, Nguyen Tat Thanh University, Ho Chi Minh City 700000, Vietnam; 10 Institute for Global Health Innovations, Duy Tan University, Da Nang 550000, Vietnam; 11 Department of Lower Limb Surgery, Vietnam-Germany Hospital, Hanoi 100000, Vietnam; 12 Institute for Preventive Medicine and Public Health, Hanoi Medical University, Hanoi 100000, Vietnam; 13 Bloomberg School of Public Health, Johns Hopkins University, Baltimore, MD 21205, USA; 14 Department of Psychological Medicine, National University Hospital, Singapore 119074, Singapore; 15 Department of Psychological Medicine, Yong Loo Lin School of Medicine, National University of Singapore, Singapore 119228, Singapore; 16 Institute for Health Innovation and Technology (iHealthtech), National University of Singapore, Singapore 119077, Singapore

**Keywords:** risk for depression, older adults, rural, Vietnam, mental health services

## Abstract

(1) Background: Major causes of the burden of disease in older persons include mental disorders and neurological diseases, such as depression. This study aims to explore the prevalence of older people at risk for depression and identify the factors associated with this risk in rural Vietnam. (2) Methods: A cross-sectional study was conducted in Soc Son, Hanoi with 523 community dwelling elders aged 60 and over. Face-to-face interviews were performed to collect data about socioeconomic status, risk for depression, health status, and health utilization. The Geriatric Depression Scale-4 items (GDS-4) was used to assess the risk for depression occurrence. Multivariable logistic regression was employed for determining the factors associated with the risk for depression. (3) Results: Among 523 participants, there were 26.4% of participants at risk for depression. The proportion of females at risk for depression (29.0%) was significantly higher than males (20.4%). Differences were found in economic status (near poor group had higher risk for depression compared to the poor group) (*p* < 0.01). Older adults living with spouse/partner, living in near-poor household, and suffering pain/discomfort were all more likely to be at risk for depression. (4) Conclusions: Being female, living in a near poor household, being in pain or experiencing discomfort are all factors strongly correlated to high risk for depression. These findings highlight the urgent need for additional research among Vietnamese community-dwelling older people.

## 1. Introduction

Improved life expectancies due to economic development and better living conditions have dramatically shifted demographic structures worldwide. In 2050, 17% of the world’s population is expected to be aged 65 or older [1]. The unprecedented growth of an aging population is posing a significant challenge to healthcare systems worldwide, including Vietnam [2]. Vietnam’s population has been defined as an aged population since 2012, and it is forecasted to be one of the fastest aging countries in the world [1]. By 2049, one in every four people will be 60 years or older, accounting for 25% of the country’s population [1]. Unfortunately, research has found that having a higher life expectancy does not equate to individuals living healthier, especially in their old age [3,4]. Vietnam, like many of its counterparts, will face numerous challenges as it prepares its healthcare system to support the unmet needs of its older people.

Despite sharing similar characteristics to other countries, the experiences of older adults in Vietnam have been uniquely shaped by a long history of war, migration, losses, and changes in traditional cultural and social norms [5]. Economic development post-war has also led to a shift in traditional family structures, with women working more outside of the home and having fewer children—leading to a diminished capacity in providing care for aging parents [5]. This has led to an increased awareness of the need to develop policies and programs within the health sector to advance the health and wellbeing of older persons in Vietnam [6].

One particular area of outstanding need is around developing a comprehensive set of policies and programs to manage the mental health needs of its aging population [1,5]. Major causes of the burden of disease in older adults include mental disorders, and neurological diseases such as depression was the eighth cause [7]. The burden of disease from neurological and mental illness is measured by years of life lost, mainly due to a disability, with depression disorders making up 17% of the burden in Vietnam [1]. Depression often results in decreased quality of life, limits one’s ability to perform self-care activities, and reduces the social connectedness of an individual [8]. If detected early, treatment and avoidance were found to be effective in older adults [9]. Additionally, there is a healthcare cost associated with depression; older individuals with depression had approximately 50% higher cost of medical services, after adjusting for age, sex, and chronic medical illnesses [10].

Despite the increased attention on mental health and the rapidly growing population in Vietnam, there are still limited studies around the prevalence of depression among this population, particularly for individuals in rural environs. This study aims to explore the prevalence of older people at risk for depression and identify the factors associated with this risk in rural Vietnam. At the local grassroots level, Vietnam has not yet developed a standard guideline on screening for early detection of depression [1]. The absence of a standard guideline on screening for early detection of depression in Vietnam has been considered one of the major difficulties/shortcomings of mental healthcare in the country. Exact reasons for such absence, we suspect, relate to a lack of information on mental health within the Ministry of Health. This work is critical to ensure that health care workers are well informed on what associated factors to screen for and is equipped with the tools needed to adequately identify older individuals at risk for depression. Early detection and proper screening to identify the prevalence of older people at risk for depression are critical for appropriate intervention and prevention. In an era where the Vietnamese government is shifting its healthcare focus to prevention, understanding the risks of depression for older adults is critical towards advancing that goal.

## 2. Materials and Methods

From February to April 2017, a cross-sectional survey was performed in Soc Son, Hanoi—a rural district where the population is about 316,000, with 523 older people who (1) were aged from 60 years old or older, (2) were able to understand and answer the questions, and (3) were willing to enroll in the study. These individuals were randomly selected from a list of older individuals residing in the study setting. After selecting the participants, with the local health professionals’ support, we contacted and invited them to go to the commune health centers near their living locations for the face-to-face interview. Collecting data via interviews was meant to ensure a high response rate and better quality of data, especially since the participants were older people from rural areas.

Each interview was conducted in 20–25 min by well-trained undergraduate medical and nursing students at the Hanoi Medical University. We developed a structured questionnaire to collect the data about:

Socioeconomic information: Socio-demographic and behavior characteristics included age, sex, occupation (retired/freelancers/farmers/others), having caregiver or not, who the caregiver was, poor status (based on Government classification [11]), and having health insurance or not (the current coverage of health insurance in Vietnam is about 80%).

Risk for depression: The Geriatric Depression Scale-4 items (GDS-4), which has been used widely among Vietnamese population but was not published, was used to assess the risk for depression among our participants: (1) Are you basically satisfied with your life? (Yes/No), (2) do you feel that your life is empty? (Yes/No), (3) are you afraid that something bad is going to happen to you? (Yes/No), and (4) do you feel happy in most of your life? (Yes/No). “Yes” answers for questions 2 and 3, and “No” answers for questions 1 and 4 scored 1 point, resulting in a range score from 0 to 4 points. If people had 4 points, they were classified as being at risk for depression [12]. The response rate was 100% (all those interviewed answered).

Other covariates: In this study, we employed four items from EuroQol-5 dimensions-5 Levels (EQ-5D-5L) including mobility, self-care, usual activity and pain/discomfort with 5 levels from “no problem” to “extreme problems” [13]. These items have been translated into Vietnamese and validated elsewhere [13,14]. We also asked patients to report their history of specific diseases common to the older adults (stroke/diabetes/Parkinson’s/high blood pressure), health service utilization in the last 12 months (including inpatient, outpatient, and regular health checkup services), current smoking and alcohol status, and whether they reported that doing physical activities at least 150 min per week such as walking and playing sports in the last 30 days.

Data analysis: *p*-value less than 0.05 was used to define statistical significance. Chi-squared test and *T*-test were employed to measure the differences in socio-economic status, health status, behaviors, and health service utilization between people at risk for depression and their counterparts. Multivariable Logistic regression was used for determining factors that were associated with the risk for depression. The regression model was combined with stepwise forward selection strategies, using the *p*-value of log likelihood of less than 0.2 for selecting variables, to build the final reduced model. Data were analyzed using STATA 12.0 software (Stata Corp. LP, College Station, TX, USA).

Ethical approval: The study protocol was received approval from the IRB of National Geriatric Hospital (Reference: No 35; 16 January 2017). Participants provided written informed consents if they agreed to participate in this study. They could withdraw from the study anytime. Their data were secured, and only the principal investigator could access these data.

## 3. Results

Among 523 participants, most respondents were female (70.0%), retired (42.5%), or farmers (43.2%), living with spouse/partner (63.2%), having a caregiver (96.2%), and having health insurance (84.3%). The mean age of participants was 71.0 (SD = 8.2) years old. There were 26.4% of participants who were at risk for depression, while 88% of them described their current situation as hopeless. (Table 1).

Table 2 shows that the proportion of females at risk for depression (29.0%) was significantly higher than that of males (20.4%) (*p* = 0.04). Differences were also found in economic status (*p* < 0.01). The “near poor” group had a higher depression risk rate (49.0%) than the poor (27.1%).

Table 3 shows that the risk for depression was significantly higher among older Vietnamese people with difficulty in mobility and pain/discomfort, as well as those having hypertension (*p* < 0.05). Meanwhile, no differences were found between those being at risk for depression and without risk for depression in other characteristics (*p* > 0.05).

Table 4 shows that living with spouse/partner, living in near poor household, and suffering pain/discomfort were more likely to be at risk for depression.

## 4. Discussion

Our study found that the prevalence of individuals 60 years or older at risk for depression was at 26.4%. In addition, the majority of participants described their situation as hopeless. These results are supportive of the increases in the prevalence of Vietnamese people at risk for depression but not conclusive. Previous studies have looked at prevalence rates of depression among older people in Vietnam, with a few studies focused on populations in rural communities. The prevalence of depression among older people has varied, but one study found at rate of 17.2% for older community-dwelling adults living in rural Vietnam (8). However, there is a lack of data in the field indicating prevalence rates of individuals at risk for depression. Therefore, further studies in this population are needed to confirm these findings.

Among the socio-demographic variables that were analyzed, females had a significantly greater risk for depression at 29% (*p* = 0.04), compared to their male counterparts (20.4%). This observation is in line with a number of studies that have indicated that women are at a higher risk for depression [15,16], and this holds true for women from similar countries as well as for women from rural communities [17,18,19]. Research has shown that women are more prone to and vulnerable to negative exposure to life stressors [20]. It has been estimated that 58% of women in Vietnam suffer from at least one type of domestic violence or abuse in their lifetime [21]. Additionally, older women in Vietnam have gone through long periods of wars, lived through life after the wars on limited resources, have been subjected to traditional gender beliefs, and were provided with less educational opportunities [20]. The multiple types of life stressors were found to be directly impacting mental health and linked with depression [22,23].

The findings align well with another observation we made on the data. A multivariate regression analysis showed that, in relation to marital status, those living with a spouse/partner compared to those who are single (*p* < 0.05), were more likely to be at risk for depression. The common occurrence of physical and sexual violence experienced by rural women in Vietnam among married couples might shed light as to the reason for the association of marital status to risk for depression [22]. Research is still unclear on why individuals who are married and/or have more social support are more likely to have depressive symptoms [5]. There are a number of studies that indicate marital status is a protective factor for depression in older people [24,25]. This finding is in strong contrast to past research and findings that support the beneficial effects of having a social support system [25]. One reason for these contrast findings include the population size, cultural norms, gender norms in the specific society being studied, definition of depression, and a number of other factors that might influence the correlation.

No significant differences were found in other factors such as occupation, caregiver type, health insurance coverage, or age. However, risk for depression was found to be associated with economic status. Particularly, older people who were living in near poor households had a significantly higher risk for depression (*p* < 0.01) compared to those living in poor or non-poor households. A majority of the older Vietnamese people living in rural communities have low income; approximately 60% have reported not having enough income to cover their basic and medical needs [1]. Poor older people have more disabilities and less access to the necessary health services that would allow them to enjoy a higher quality of life [26,27]. On the other hand, it is worth mentioning that people classified as poor in Vietnam—having a monthly income of less than 700,000 VND or US $30 [11] received subsidy and financial assistance from the Government to cover most basic living necessities (housing, water, and electricity, health insurance, for instance), while those classified as near-poor (living on income of less than 1,000,000 VND or US $50 but higher than US $30 [11]) did not. This would potentially be one of the main reasons for the near-poor participants having a significantly higher likelihood of having depression. Nonetheless, lower socioeconomic status is generally correlated with having a higher risk for depression, the near-poor group tends to care more about their living expense while the poor-group receives funds from the government [11]. A meta-analysis on socioeconomic inequalities in depression, which was conducted on a number of studies including 51 prevalence studies, supports the finding that individuals with a lower socioeconomic status have higher odds of being depressed (odds ratio = 1.81, *p* < 0.001) [26].

This study found that older Vietnamese people with difficulties in mobility, particularly those having pains and discomforts (OR = 1.92, *p* < 0.05), were at significantly higher risk for depression. This association is aligned with previous studies that have shown, through a systematic review, that impairment of activities and difficulties in mobility was associated with depressive symptoms [28]. A longitudinal analysis also confirmed the strong association between pain and depression in Dutch older adults after adjusting for gender and age [29]. Pain and discomfort are unfortunately commonly observed in the older population. However, left untreated or unattended in an older adult, it can lead to detrimental consequences such as adversely impacting mood, social functions, quality of life, and mobility [30,31]. These factors have been documented for risk for depression in the older population experiencing pain and discomfort [30,32]. These results illustrate the importance of treating pain and discomfort that arise through the aging process for an older adult [32]. Pain treatment and management can alleviate the suffering of older people and potentially reduce the risk of developing depression.

The data also highlights that older people having a comorbidity of hypertension (*p* < 0.05), were at higher risk for depression. The relationship between hypertension and depression remains unclear and controversial despite the many studies that have researched this topic [33]. In 2015, Long et al. conducted a meta-analysis to consolidate findings around this subject and provide a clearer understanding of the relationship; however, results were inconclusive on hypertension as a risk factor for depression [33]. It might be worth noting that the studies in the meta-analysis might have varied in population size, demographics, locations, and a number of other differences—and might not be representative of the rural older Vietnamese population we are studying. A study on the association between hypertension and depression among older people in China led to the conclusion that hypertension is a modifiable risk [34]. The association of hypertension with risk for depression was dependent on other conditions such as illiteracy, low income, living in rural areas, and being single [34]. Additional research will help validate findings for the risk factors associated with depression.

Clear associations were found between variables such as gender, economic status, level of mobility, and being at a higher risk for depression. The identified associated factors can be used by healthcare leaders and practitioners to help identify older adults who are most at risk for depression. These results, along with findings from similar studies as mentioned above, point to a need for additional research, particularly focused on Vietnamese older people in rural communities. Health professional and programs should provide reliable screening for the risk of depression. This will allow for more informed health care and services for older adults who are at risk or suffering from depression. Depression places an undue burden on the country’s healthcare system as well as decreases the quality of life for an individual experiencing it. Therefore, identification of individuals at risk is needed to get them the care and services they need, prevent suffering and advance the quality of life for Vietnamese older adults in living in rural communes.

## 5. Limitations

There are several limitations to this study. Given the cross-sectional nature of the surveys, only associations rather than causal relationships between the risk for depression and associated factors could be determined. We were unable to identify with certainty the temporality between some of the variables studied in this study with the risk for depression because the sequence of events is uncertain. For example, it might be difficult to assess whether hypertension puts older adults at a higher risk for depression or if the risk for depression leads to individuals having hypertension.

Due to the study design, the surveys were also susceptible to various biases such as social acceptability bias, responder bias, and interviewer bias. Additionally, because we only employed four questions in the questionnaire used to screen for the risk for depression, we were limited in the scope and might not have accounted for factors outside the scope of the study. Lastly, there are limitations to the generalizability of the findings given that the study was conducted in a single rural community; the sample might not be representative of the elderly population of rural Vietnam in its entirety.

## 6. Conclusions

All older adults should have an opportunity to go through a healthy and normal aging process, without being impaired by the development of depression. The capacity for identifying and screening for at risk individuals for depression in rural Vietnam is currently inadequate. This poses a challenge for the government to provide the necessary services to respond to the growing aging population and its mental health needs. Proper screening for at risk older adults can prove beneficial from a prevention and early treatment perspective.

Being female, living in a near poor household, being in pain or experiencing discomfort are all factors strongly correlated to high risk for depression. Health professionals and policymakers should take these factors in consideration when they design healthcare programs to advance the healthy aging of older persons in rural communes. Addressing these factors might potentially prove advantageous in reducing the risk of depression. The study highlights the urgent need for additional research to explore further and understand the prevalence of those at risk for depression among Vietnamese community-dwelling older persons in rural communities. The findings from this work can be used to generate additional hypotheses and serve as a foundation for future research.

## Figures and Tables

**Table 1:** Risk for depression among older people based on the Geriatric Depression Scale-4 items (GDS-4).

Characteristics	N	%
**Assess the Risk for Depression**		
Satisfied with life	104	19.9
Empty life	74	14.2
Hopeless	465	88.0
Happiness	44	8.4
Risk for Depression *		
No	385	73.6
Yes	138	26.4

* Note: “Yes” answers for question 2, 3, and “No” answers for question 1, 4 scored 1 point, resulting in a range score from 0 to 4 points. If people had 4 points, they were classified as being at risk for depression.

**Table 2:** Risk for depression according to socio-economic status of respondents.

Characteristics	Risk for Depression				Total		*p*-Value
	No		Yes				
	n	%	n	%	n	%	
**Gender**							
Male	125	79.6	32	20.4	157	30.0	0.04
Female	260	71.0	106	29.0	366	70.0	
**Occupation**							
Retired	171	77.0	51	23.0	222	42.5	0.12
Freelancers ^+^	18	58.1	13	41.9	31	5.9	
Farmers	166	73.5	60	26.6	226	43.2	
Others ^++^	30	68.2	14	31.8	44	8.4	
**Marital Status**							
Single	20	76.9	6	23.1	26	5.0	0.10
Live with spouse/partner	252	76.4	78	23.6	330	63.2	
Divorced/separation/widow	112	67.5	54	32.5	166	31.8	
**Caregiver**							
Wife and children	339	74.2	118	25.8	457	90.9	0.13
Grandchildren	28	75.7	9	24.3	37	7.4	
Others	4	44.4	5	55.6	9	1.8	
Economic Status *							
Poor	35	72.9	13	27.1	48	10.3	<0.01
Near poor	26	51.0	25	49.0	51	10.9	
Non-poor	277	75.5	90	24.5	367	78.8	
**Health Insurance**							
No	62	75.6	20	24.4	82	15.7	0.66
Yes	323	73.2	118	26.8	441	84.3	
	**Mean**	**SD**	**Mean**	**SD**	**Mean**	**SD**	
**Age**	71.3	8.2	70.3	8.2	71.0	8.2	0.23

^+^ Those working jobs without formal labor contract and those who are self-employed. ^++^ Including homemaker and builder. * Based on the Government’s classification. Abbreviation: SD: Standard Deviation.

**Table 3:** Health status, health utilization, and behavior (smoking/drinking/doing physical activities) of respondents.

Characteristics	Risk for Depression				Total		*p*-Value
	No		Yes				
	n	%	n	%	n	%	
**EQ-5D-5L**							
Difficulty in mobility *	181	68.8	82	31.2	263	50.3	0.01
Difficulty in self-care *	89	69.0	40	31.0	129	24.7	0.17
Difficulty in usual activities *	163	69.4	72	30.6	235	44.9	0.04
Pain/Discomfort *	265	70.1	113	29.9	378	72.3	<0.01
**Comorbidities**							
Stroke *	9	90.0	1	10.0	10	1.9	0.24
Anemic cerebral ischemia *	18	64.3	10	35.7	28	5.4	0.25
Diabetes *	18	75.0	6	25.0	24	4.6	0.88
Chronic lung disease *	24	68.6	11	31.4	35	6.7	0.48
Arthritis *	106	70.7	44	29.3	150	28.7	0.33
Hypertension *	134	79.3	35	20.7	169	32.3	0.04
**Health Service Utilization in the Last 12 Months**							
Inpatient	54	67.5	26	32.5	80	15.3	0.18
Outpatient	194	74.9	65	25.1	259	49.5	0.51
Regular health check-up	276	73.8	98	26.2	374	71.5	0.88
**Smoking Status**							
No	343	72.5	130	27.5	473	90.4	0.12
Former smoker	21	91.3	2	8.7	23	4.4	
Current smoker	21	77.8	6	22.2	27	5.2	
**Alcohol Drinking Status**							
No	302	72.6	114	27.4	416	79.5	0.58
Former drinker	11	78.6	3	21.4	14	2.7	
Current drinker	72	77.4	21	22.6	93	17.8	
**Doing Daily Physical Activities in the Last 30 Days**							
Yes	238	75.8	76	24.2	314	60.0	0.17
No	147	70.3	62	29.7	209	40.0	

* Test for having/not having particular health problems. Abbreviation: EQ-5D-5L: EuroQOL-5 Dimensions-5 Levels.

**Table 4:** Multivariate regression of factors associated with risk for depression.

Characteristics	Risk for Depression	
	OR	95% CI
**Age**	0.97 *	0.94; 1.00
**Marital Status (vs Single)**		
Live with spouse/partner	1.65 **	1.02; 2.68
**Economic Status (vs Poor)**		
Near poor	2.62 ***	1.36; 5.03
**Having Health Insurance (Yes vs. No)**	1.63	0.87; 3.03
**Smoking Status (vs No)**		
Former smoker	0.33	0.07; 1.51
**Having Problems in**		
Usual activity (Yes vs. No)	1.36	0.86; 2.16
Pain/Discomfort (Yes vs. No)	1.92 **	1.10; 3.33
**Having Comorbidity**		
Hypertension	0.71	0.43; 1.17

*** *p* < 0.01, ** *p* < 0.05, * *p* < 0.1; Abbreviation: OR: Odds ratio; CI: Confident interval.
